# The mechanisms and management of persistent postsurgical pain

**DOI:** 10.3389/fpain.2023.1154597

**Published:** 2023-07-06

**Authors:** Alice M. Fuller, Sabah Bharde, Shafaq Sikandar

**Affiliations:** William Harvey Research Institute, Charterhouse Square, Queen Mary University of London, London, United Kingdom

**Keywords:** PPP, risks, pre-clinical models, mechanisms, prevention

## Abstract

An estimated 10%–50% of patients undergoing a surgical intervention will develop persistent postsurgical pain (PPP) lasting more than 3 months despite adequate acute pain management and the availability of minimally invasive procedures. The link between early and late pain outcomes for surgical procedures remains unclear—some patients improve while others develop persistent pain. The elective nature of a surgical procedure offers a unique opportunity for prophylactic or early intervention to prevent the development of PPP and improve our understanding of its associated risk factors, such as pre-operative anxiety and the duration of severe acute postoperative pain. Current perioperative pain management strategies often include opioids, but long-term consumption can lead to tolerance, addiction, opioid-induced hyperalgesia, and death. Pre-clinical models provide the opportunity to dissect mechanisms underpinning the transition from acute to chronic, or persistent, postsurgical pain. This review highlights putative mechanisms of PPP, including sensitisation of peripheral sensory neurons, neuroplasticity in the central nervous system and nociceptive signalling along the neuro-immune axis.

## 1. Introduction

Acute pain after surgery typically resolves within 1–2 weeks with appropriate control therapies (1). However, for some patients, acute postsurgical pain continues beyond the normal time for tissue healing and transitions into a “chronic” or persistent pain state. Persistent postsurgical pain (PPP) is pain that is localized to the surgical field or to an innervation territory of a nerve situated in the surgical field, or to a dermatome after surgery in deep somatic or visceral tissue and persists for at least 3–6 months (2). A universally recognized definition of PPP is essential to guide future research strategies. The first definition was proposed by Macrae and Davies in 1999 (3):

1) Pain must develop after a surgical procedure
2) The pain is of at least 2 months duration
3) Other causes for pain have been excluded
4) The possibility that the pain is from a pre-existing condition has been excluded

One shortcoming of the original definition was the assumption that PPP is acute postsurgical pain that persists over a defined period. However, empirical studies show that a proportion of patients are pain-free or have mild pain in the acute post-operative period, with different sensations emerging or new pain onset weeks or months after the initial surgical insult (4, 5). The lack of correlation between early and late pain outcomes after surgery highlights the necessity to understand the biopsychosocial risk factors for developing PPP. Furthermore, excluding pre-existing pathologies or undiagnosed pain syndromes may be a confounding factor in reported studies. Schug et al. (6) described a reduction from 40% to 18% in the prevalence of PPP when only moderate or severe pain around the area of surgery was taken into consideration. But this dropped to 6% when patients who had pre-existing pain before surgery were excluded, highlighting the importance of considering pre-existing pain and its characteristics.

To enable adequate identification, diagnosis and therapy, PPP is now included in the *International Classification of Diseases (ICD-11)* (7)*.* The updated definition postulates a more global measure of the impact of pain on quality of life for the individual (2, 8). It also considers pain severity (to identify chronic pain), and encompasses 3 dimensions: pain intensity, pain-related distress, and pain-related functional interference (9).

## 2. Incidence and prevalence

More than 300 million people worldwide undergo surgery each year. Although acute postsurgical pain is expected and can be well managed, even minor interventions are linked with the risk of developing PPP (10, 11). The development of persistent pain following surgical procedures remains a significant clinical problem that has a debilitating impact on postsurgical rehabilitation and quality of life. In the UK alone, severe PPP (defined as a score >5 on the Numeric Rating Scale (NRS) (7)) affects 2%–10% of adults undergoing surgery (12) and corresponds to at least 140,000 patients per year presenting with disabling pain. The global incidence of PPP varies depending on the type of surgery (5%–85%; see Table 1) and the median incidence of chronic pain at 6–12 months post-surgery is 20%–30%, with a small decrease over time (7, 15). The wide intra-variability in PPP incidence is predominantly due to methodological details caused by varying methods of data collection and lacking consensus in the definition of PPP (16). The incidence of PPP for different types of surgery are visible in Table 1.

**Table 1:** Incidence of postsurgical pain in a number of surgical interventions.

Type of surgery	Incidence of persistent postsurgical pain (%)
Abdominal surgery	17–21
Amputation	30–60
Caesarean section	6–55
Coronary bypass	30–50
Cholecystectomy	3–56
Craniotomy	7–30
Dental surgery	5–13
Hip arthroplasty	7–23
Inguinal herniotomy	5–63
Knee arthroplasty	13–14
Mastectomy	11–57
Melanoma resection	9
Sternotomy	7–17
Thoracotomy	5–71
Vasectomy	0–37

Data adapted from multiple studies (7, 13, 14).

Most clinical studies that aim to quantify pain outcomes following a surgical procedure focus on data composed either within a single institution or on a national level. Published studies based on single-site recruitment typically use patient data from the perioperative period, are often limited to one specific type of surgery and utilise small sample sizes (15, 17). On the other hand, nationwide studies constitute larger sample sizes, but often report retrospective data that can hinder the possibility to accurately assess temporal changes in the development of postsurgical pain and patient reported outcomes. More recently, large prospective international studies have begun to employ standardized methods for recording surgical and other perioperative characteristics (e.g., use of analgesics), as well as standardized questionnaires to gauge psychological factors (e.g., mood and catastrophizing) (18–20).

A major challenge in patient recruitment is distinguishing PPP from continuing pre-existing pain; a seminal study by Hofer et al. (21) emphasizes the need for patients with pre-existing pain to be evaluated more comprehensively, both pre- and post-surgery. Based on different patient outcomes, the findings of this study suggest that the use of cut-offs in categorizing patients into groups “PPP” or “no PPP” does not meet the needs of an individual subject, but rather that there is a transition from one group to the other. The results corroborate findings from other studies, where there are subgroups of patients that are “mixed” with milder pain symptoms, but little to no functional interference, or with essentially no pain and moderate interference with functional tasks (in addition to patients who experience pain with functional limitations). The comprehensive assessment of pain and functional interference led to lower PPP rates than previously reported. Pre-existing chronic pain, preoperative opioid medication and surgery type were also correlated with patient-reported pain outcomes 12 months post-operatively (21). In another population-based study, 18% of patients described moderate-to-severe pain at the surgical site 3–36 months after surgery (22). However, if patients who had similar pain ratings pre-surgery were excluded, only 10.5% would be classified to have PPP, while if patients with any pain before surgery were excluded, this drops to 6.2%. A change of pain characteristics or sensory disturbances, or location or spatial distribution pre-surgery can be additional diagnostic criteria, as 74.1% reported a change in the “kind of pain” compared to pre-operative pain (22). However, these postsurgical changes in pain characteristics are not addressed in the ICD-11, and questionnaires do not always consider deviations in temporal patterns and type of pain, leading to the potential misclassification of patients with PPP. Moreover, the common use of “unidimensional tools” in clinical studies, such as the sole use of the visual analogue scale (VAS), does not reflect the multi-faceted nature of the pain experience that can be better captured in combination with functional and psychometric measurements (23).

## 3. Risk factors for postsurgical pain

Identifying patients that may be at risk of PPP is a promising start for adequate postsurgical pain management. Current multimodal strategies to avert pain seem inadequate, mainly due to a lack of targeted approaches to personalized pain management (24–26). Improving acute pain after surgery is strongly dependent on identifying, preventing, and quantifying postsurgical pain in a timely manner; and recognizing early postsurgical pain may prevent pain from becoming chronic (27). Risk factors for PPP can be broadly identified by temporal characteristics in the pre-, peri- and post-operative periods and cover six general domains: genetic, demographic, psychosocial, pain, clinical and surgical factors. Whilst many studies have contributed to growing evidence of risk factors such as age, higher body mass index (BMI), psychological traits like depression and anxiety, and sex, besides the type of surgical intervention itself (24, 25), no single factor appears to dominate, and the question of causation remains unanswered. For example, less than 20% of the complete risk of chronic pain can be predicted by the severity of acute postsurgical pain (26). However, it is conceivable that the cumulative risk may become substantial in patients who present with multiple risk factors. Prediction models would be helpful if clinically applied for risk stratification and tailoring appropriate treatments for patients at most risk of developing PPP. This section will describe in detail some of these risk factors that have been addressed in clinical studies and are summarized in Table 2.

**Table 2:** Summary of factors contributing to higher risk of developing postsurgical pain.

Risk factor	Considerations
Patient demographics	Age, sex
Genetics	Multiple mutations and SNPs
Psychological	Depression, psychological vulnerability, stress, anxiety and catastrophising
Pain	Pre-operative acute/chronic pain, preoperative use of analgesics, decreased CPM, increased temporal summation and increased sensitivity to experimental pain
Surgical	Type of surgery, nerve injury, longer duration of surgery
Clinical	Comorbidities, disabilities, radiotherapy, or chemotherapy

CPM, conditioned pain modulation; SNPs, single nucleotide polymorphisms.

### 3.1. Pre- and peri-operative factors

#### 3.1.1. Pre-existing pain

*Pre-existing pain* is an established and leading risk factor for PPP. Many lines of evidence demonstrate that the presence and intensity of preoperative chronic pain, the intensity of acute postsurgical pain, the time spent in severe pain after surgery, pain intensity in the weeks after surgery, and pain in other body parts reliably predict the extent of PPP across a variety of surgical procedures (25, 27). In a cohort of patients undergoing abdominal surgery, higher pain ratings immediately before surgery and the occurrence of chronic pain before surgery (lasting >6 months) independently predicted moderate to intense pain in the acute postsurgical period (28). Similarly, preoperative pain at or near the operative site is a useful tool to predict chronic pain after hernia and amputation surgery (29–31). Furthermore, the total number of painful sites (pre-operatively) corresponds to the probability of developing PPP (25, 27). Methodologically, it is important to measure pain status preoperatively, as retrospective recall of preoperative pain may be biased by presence and intensity of current pain.

More recently, Luedi *et al*. used pressure point thresholds (PPT) pre-, peri- and post-operatively on 128 subjects undergoing anorectal surgery, in addition to analysing the postsurgical analgesic medication consumption as a secondary outcome (32). This study demonstrated that patients with lower thresholds preoperatively had higher postsurgical pain scores on day 3 and at 4 weeks, but not day 1 after surgery. Thus, PPT testing may serve as an aid for identifying patients at risk of developing severe postsurgical pain. Furthermore, various studies have demonstrated the impact of chemo/radiotherapy on the chronification of pain, where either pre- or post-surgical exposure to chemotherapeutic medications have been shown to cause painful neuropathy, and in some patients, worsen PPP (33, 34). This may be due to the possible involvement of mitochondrial RNA dysregulation caused by chemotherapy-induced cellular toxicity (35–37).

#### 3.1.2. Demographic risk factors

Regarding *age*, older patients tend to have a lesser risk of developing PPP compared to younger patients (27, 38–40). This is opposed to findings in other chronic pain states where increased age can predispose one to higher rates of chronic pain (41). Female *sex*, which is a generalized risk factor for developing severe acute and chronic pain is also a risk factor for PPP (27, 38). Another recent report identified factors related with acute postsurgical pain trajectories and whether this can predict chronic postsurgical pain in a cohort of patients undergoing gastrointestinal surgery (42). In the acute pain trajectory, female sex and preoperative anxiety were predictive factors of chronic pain 6 months postoperatively, whereas age was a predictive factor for chronic pain 3 months after surgery. Overall, the mechanistic basis of sex differences in the prevalence of PPP are unknown but putative links have been made to genetic, psychological, socio-cultural, and pharmacokinetic differences between cis men and cis women (43).

In relation to members of the transgender community, many experience distress, depression and anxiety which may contribute to the development of persistent pain states, such as fibromyalgia (44). Whether or not transgender individuals are more prone to PPP is still unknown due to the underrepresentation of minority groups in clinical research, although one recent study demonstrated 27.4% of trans-men undergoing bilateral mastectomy reported either unilateral or bilateral persistent pain after surgery (45). Studies with larger cohorts and documented medications such as hormone therapies are required to identify susceptibility of transgender individuals to PPP.

#### 3.1.3. Genetics

Chronic pain is currently regarded a complex trait and has a heritability in the range of 30%–70%, therefore it is unsurprizing that PPP would have genetic risk factors. However, no single genetic cause has been identified (46, 47). Both single nucleotide polymorphisms (SNPs) and epigenetic changes can affect various functions in the inflammatory and stress responses. Pain susceptibility is known to be influenced by several genes, and the genetic basis of the individual physiological response to injury and pain management in post-operative patients is exemplified by the salient variation in a study where equivalent numbers of patients undergoing thoracotomies for lung cancer (up to 25% in a cohort of >500) report either low or high pain scores already in the first 2 post-operative days (48). Most human genetic studies focused on postsurgical pain have largely investigated polymorphisms in candidate genes. One study showed that the severity of PPP is correlated with the mRNA expression of signal transduction genes (49). The presence of PPP has also been associated with mutations in genes encoding catechol-O-methyltransferase (an enzyme in the monoaminergic pathway influencing pain inhibition) and *Oprm1* encoding the μ-opioid receptor (50).

The first genome-wide association study identified single-nucleotide polymorphisms associated with the development of PPP 3 months after surgery based on two independent cohorts (51). A discovery cohort of patients undergoing hysterectomy was utilized and potential candidates were further explored in a replication cohort of orthopaedic and abdominal surgeries to verify findings. One locus (*NAV3*) was associated with PPP in the replication cohort, and two loci (*CRTC3 and IQGAP1*) were significantly associated with PPP in the meta-analysis of both cohorts (51). Moreover, a meta-analysis by Chidambaran et al. (2020) identified significant gene variants for 26 genes involved in neurotransmission, pain signalling and immune responses linked with PPP in 21 human genetic studies (47). This study establishes the role of genetic factors with different functions in PPP, and that single genetic factors have small effect sizes in explaining PPP pathophysiology. The heterogeneity in surgical cohorts, population, outcome definitions and the number of available studies evaluating these variants restricts further analysis. Thus, there is a need for large-scale, homogeneous, replication studies to validate candidate genes and recognise the fundamental biological networks for underpinning PPP. One study that is expected to finish in 2025 combines several outcome measures in a large cohort (10,000 patients) and will enable stratification for patients based on pain outcomes using genetic, demographic and clinical factor prediction models (52).

#### 3.1.4. Psychosocial risk factors

Chronic pain is now commonly considered as a “sociopsychobiomedical” issue, which supports the paradigm shift in the approach to investigating and reporting PPP (53). Psychosocial risk factors for the development of PPP entail both cognitive behavioral and emotional concepts, including preoperative anxiety and trait anxiety, as well as fears associated with impending surgery, and the concept of catastrophizing (54). Furthermore, negative cognitive-affective states including perioperative depression (55, 56), anxiety (56–59), pain catastrophizing (18, 59) and post-traumatic stress symptoms (19, 60) predict the onset of PPP. The protective role of positive psychological factors has also been addressed, such as resilience and optimism, largely interpreted as the capacity to respond effectively to risk or adversity (20, 61). Furthermore, a study by Armstrong et al. (62) indicated that anxiety, current smoking, other psychological conditions and preoperative opioid use were significantly associated with higher reported pain scores before surgery, as well as 24–48 h post-operatively.

#### 3.1.5. Use of pre-operative and peri-operative analgesics

Although pre-operative opioid consumption is warranted by the potential to effectively control acute postsurgical pain and patient function, it has also been surprisingly shown to be a risk factor for PPP (in ca. ∼25% of surgical interventions) (60, 61, 63, 64). For example, intraoperative remifentanil dosage is highly correlated with increased incidence of PPP up to 1 year after cardiac surgery (65, 66). This may be in part due to opioid-induced hyperalgesia (61, 67) as some patients who are already on long-term opioids may have developed this phenomenon, subsequently leading to poorly controlled postsurgical pain and increased opioid requirements (68, 69). Several studies address the influence of preoperative opioid consumption on PPP (66, 70), notably Keller et al. (71) demonstrated that 48% patients consuming opioids before thoracotomy had chronic pain post-surgery, in contrast to 5% of patients not consuming opioids. Furthermore, opioid administration during surgery may activate N-methyl-d-aspartate (NMDA) receptors and/or glial cells leading to sensitisation of peripheral and central nervous system centres (46, 61, 72, 73).

### 3.2. Intra-operative and post-operative factors

During the intra/post-operative periods, imminent surgical factors include the type of surgery, the surgical technique, the anatomical location, the extent of nerve injury and tissue ischemia. As an example, the degree of intercostal nerve injury is thought to be the chief element of persistent-post-thoracotomy pain (74, 75).

#### 3.2.1. Surgical intervention

Several clinical reports highlight nerve damage during surgery as one of the main factors contributing to PPP. During surgery, nerves are at risk of injury from partial or complete transection, stretching, crushing, electrical damage, entrapment, or compression (35, 74, 76–78). Nerve injury is associated with many molecular and cellular changes comprizing neuroimmune interactions (79, 80). It is entirely conceivable that duration of surgery, associated with sustained peripheral injury, could trigger changes in the central nervous system (CNS). Yet there is still relatively little data to support the notion that the more severe the surgical insult, the greater the risk of persistent pain (28, 81). Surgically-induced neuropathic pain is reported to occur in 60% of patients after limb amputation (82), in 20%–40% after mastectomy (4, 83, 84), in 20% after hernia repair (85) and in 20%–40% after thoracotomy (86, 87).

#### 3.2.2. Acute postsurgical pain

The link between acute pain after surgery and PPP has been demonstrated in some surgical cohorts, including patients undergoing hernia, breast, thoracic, and laparoscopic cholecystectomy surgery (3, 12, 88). Likewise, severe pain immediately before or after lower limb amputation predicts persistent pain in the phantom/residual limb (89). Furthermore, pain trajectories in the acute postsurgical period have revealed that trajectories with constant higher pain intensity carry the most risk (90, 91). However, the specific nature of the mechanisms and whether the relationship is causal has yet to be determined (38).

In a heterogeneous sample of patients having minor, intermediate and major surgery, Peters et al. (92) reported that surgeries lasting longer than 3 h led to increased pain and poor functional outcomes at 6 months. Severe acute postsurgical pain was also indicative of increased pain at 6 months. Other factors such as the presence and intensity of acute pain have been found to predict pain weeks and months after surgery (93). Inpatients undergoing herniotomy or total knee joint replacements, greater experimental pain ratings pre-operatively is also a recognized as a risk factor for PPP (43). Other factors are increased temporal summation, as a measure of increased excitability, and decreased conditioned pain modulation (CPM), as a manifestation of decreased inhibitory function pre-operatively.

## 4. Animal models and clinical relevance in postsurgical pain

Improving postsurgical pain management will reduce the socioeconomic impact of persistent pain and associated strain on the healthcare system. Preclinical studies provide a means of dissecting mechanisms of PPP and the aetiology of incisional pain. In this section we discuss animal models of postsurgical pain, and their limitations (94).

### 4.1. Animal models of postsurgical pain

**Figure 1:**
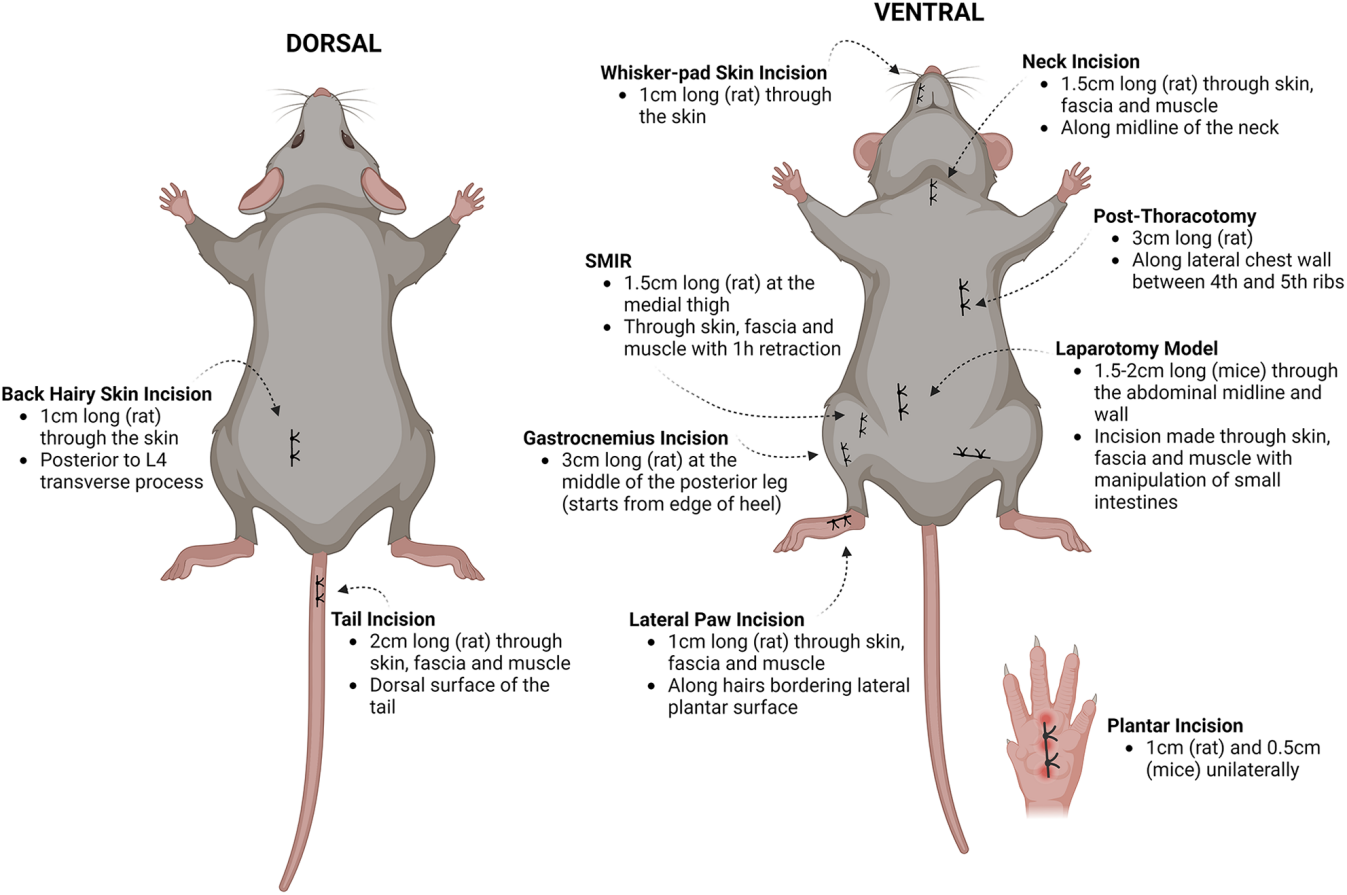
Summary of preclinical models of postsurgical pain. A series of incisional and postsurgical pain models were developed, stemming from the classical plantar incision model. Illustration modified from Pogatzski-Zahn et al. (97) created using Biorender.com. Details of the models are collated from several studies (98–103, 106, 107, 109). SMIR, skin and muscle incision retraction.

The Brennan model of post-incisional pain was the first rodent model of surgical pain and continues to be widely used (1, 95–97). This model has been adapted in terms of the depth of incision (with/without muscle injury) and the location of incision [e.g., hindpaw (98, 99), thigh (100), back (101) as well as others (102–106)]. Additional surgical pain models have since been established to mimic specific surgical procedures such as the post-thoracotomy (107) and laparotomy model (106), which can enable a better understanding of procedure-specific pain after surgery (108). Moreover, the use of prolonged tissue retraction (a method used during surgery to expose the surgery site) is captured in some models including the skin and muscle incision retraction (SMIR) model, the inguinal hernia repair model and post-thoracotomy model. These models are characterized by chronic pain behavior and may recapitulate the transition from acute to chronic pain. The variations are shown in Figure 1, and are categorized as acute, transitional, or chronic in Table 3.

**Table 3:** Cellular mediators of acute, transitional, and chronic incisional pain.

	Location	Target	Model/incision type	Species	Altered expression	Substance	Pain behavior	Ref
Acute	Spinal cord	AMPA	Plantar incision	Rats		**i.t.** or **i.p.** LY293558 (non-NMDA receptor antagonist)	**Reduced** mechanical hypersensitivity	(110)
	Spinal cord	AMPA/Kainate	Gastrocnemius incision	Rats		**i.t.** NBQX (AMPA/KA antagonist) 2 h postop **i.t.** JSTX (Joro spider toxin) (Ca^2+^ permeable non-NMDA receptors antagonist) 2 h postop	**Reduced** secondary mechanical hyperalgesia 2 h–2 days postop	(111)
	PNS	ASIC3	Plantar incision	Rats	**Increased** in ipsilateral DRG 24 h after incision	**i.pl.** APETx2 during surgery **i.t.** siRNA (once a day for 4 days preop)	**Reduced** non-evoked pain and heat hyperalgesia 24 h postop	(112)
	PNS	C5a (Complement component C5)	Plantar incision	Mice	**Increased** C5a in plantar skin from 2 to 72 h **Increased** C5aR in plantar skin from 24 to 72 h	**i.pl.** C5aR antagonist 1 h preop, then 2 h before behavior	**Reduced** thermal and mechanical hypersensitivity	(113)
	PNS	CCL17 and CCL22	Plantar incision	Mice	**Increased** CCL17 in plantar skin from 3 to 6 h **Increased** CCL22 in plantar skin from 24 to 72 h	**i.pl.** C021 (CCR4 antagonist) 1 h preop **i.pl.** C021 24 h postop **CCR4KO** (constitutive, global)	**Reduced** mechanical hypersensitivity at 1 h (not thermal) postop **Reduced** mechanical and thermal hypersensitivity 1 h after C021 **Reduced** mechanical hypersensitivity 14 h–72 h postop	(114)
	Spinal cord	GluR1 (linked to AMPA)	Plantar incision	Rats	**Increased** DH surface delivery of GluR1 at 3 h on ipsilateral side + interaction with stargazin (regulates synaptic targeting of AMPA) increased	**i.t.** Stargazin siRNA twice a day for 3 consecutive days preop	**Reduced** mechanical hypersensitivity at 3 h postop	(115)
	PNS	IL-1	Plantar incision	Mice		**IL-1rKO** **IL-1raTG** (IL-1 receptor antagonist overexpression) **s.c.** IL-1ra chronic micropump from 3 days preop OR **i.p.** 30 min before sensory testing on POD 1	**Reduced** mechanical hypersensitivity	(116)
	PNS	IL-10	Plantar incision	Rats	**Increased** in plantar skin at 4, and 48 h **Increased** in muscle from 1 h–10 days			(117)
	PNS	IL-1ß	Plantar incision	Mice	**Increased** in plantar skin from 2 to 72 h	**i.pl.** Anakinra (antagonist) 2 h preop, then daily 2 h before behavior	**Reduced** mechanical and thermal hyperalgesia	(118)
	PNS	IL-1ß	Plantar incision	Rats	**Increased** in plantar skin from 1 to 48 h **Increased** in muscle from 1 to 4 h			(117)
	PNS	IL-6	Plantar incision	Rats	**Increased** in plantar skin from 1 to 48 h **Increased** in muscle 1 h–10 days			(117)
	PNS	Na_v_1.7	Plantar incision	Mice		**i.pl** or **s.c.** Pn3a (selective Na_v_1.7 inhibitor) 24 h postop	**Reduced** mechanical hypersensitivity	(119)
	PNS	Neutrophils	Plantar incision	Mice	**Increased** in plantar skin from 2 to 72 h	**i.v.** Vinblastine sulphate 72 h preop **i.v.** Anti-neutrophil antibody 7 days and 24 h preop	**Reduced** mechanical hypersensitivity	(120)
	PNS	NGF	Plantar incision	Rats	**Increased** in plantar skin from 4 h–10 days	**i.p.** Anti-NGF antibody 1 day preop	**Reduced** non-evoked pain 4–48 h postop **Reduced** heat hyperalgesia 24–48 h **No change** to mechanical threshold	(121)
	PNS/CNS	NMDA receptors	Plantar incision	Rats		**s.c.** Ketamine perioperatively	**No change**	(122)
	PNS/CNS	Opioid receptors	Plantar incision	Rats		**s.c.** Fentanyl perioperatively	**Reduced** mechanical hypersensitivity at **Increased** mechanical hypersensitivity POD1 to POD5, and non-evoked pain D0–POD4	(122)
	PNS/CNS	Opioid receptors	Plantar incision	Rats		**s.c.** Naloxone POD 8	**No change**	(122)
	Spinal cord	PKCγ (linked to GluR1 and AMPA)	Plantar incision	Rats	**Increased** DH membrane translocation on ipsilateral side at 3 h	**i.t.** PKCγ siRNA twice a day for 3 consecutive days preop	**Reduced** mechanical hypersensitivity at 3 h postop	(123)
Transition from acute to chronic	CNS	5-HT_3_ receptor	Plantar incision + NTX (naltrexone) POD 21			**i.t.** Ondansetron (5-HT_3_ receptor antagonist) at time of s.c. NTX injection	**Prevented** NTX-induced mechanical and thermal hyperalgesia	(124)
	Spinal cord	MOR/DOR/KOR	Plantar incision	Mice		**i.t.** CTOP (MOR-selective inhibitor)/naltrinode and TIPP (DOR-selective inhibitors)/nor-BNI and LY24,56,302 (KOR inhibitors) on POD 21	**Reinstated** mechanical hyperalgesia with MOR and KOR-selective antagonists	(125)
	CNS	NMDA receptors	Plantar incision + fentanyl + naloxone on POD 8	Rats		**s.c.** Ketamine perioperatively	**Prevented** fentanyl-enhanced hyperalgesia, and naloxone-induced hyperalgesia on POD 8 Improved efficacy of morphine on POD 1	(122)
	CNS	NMDA receptors	Plantar incision + remifentanil + nor-BNI on POD 21	Mice		**s.c.** MK-801 (prior to nor-BNI)	**Prevented** nor-BNI-induced hyperalgesia	(126)
	CNS	Opioid receptors	Plantar incision + fentanyl	Rats		**s.c.** Naloxone on POD 8	**Reinstated** mechanical hyperalgesia and non-evoked pain	(122)
	CNS	Opioid receptors/KOR	Plantar incision + remifentanil	Mice		**s.c.** Naloxone/naloxone-methiodide (peripherally-restricted)/nor-BNI on POD 21	**Reinstated** mechanical hyperalgesia with naloxone and nor-BNI up to 5 months postop	(126)
	Spinal cord	PKMζ	Plantar incision + i.pl. PGE2 at day 15	Mice		**i.t.** ZIP (PKMζ inhibitor) on POD 13	**Prevented** persistent mechanical hypersensitivity induced by i.pl. PGE2 injection	(127)
Chronic	Spinal cord	IL-1ß	SMIR	Rats	**Increased** in ipsilateral DRG from 5 to 20 days	**i.t.** IL-1ra (IL-1ß inhibitor) 30 min preop and for 10 consecutive days postop	**Reduced** mechanical hyperalgesia	(128)
	Spinal cord	P2X7R	SMIR	Rats	**Increased** in ipsilateral dorsal horn from 1 to 22 days	**i.t.** BBG or A438079 (P2X7R antagonists) 30 min preop and for 7 consecutive days postop	**Reduced** mechanical hyperalgesia	(129)
	Spinal cord	p38	SMIR	Rats	**Increased** in ipsilateral DRG from 5 to 20 days	**i.t.** SB203580 (p38 inhibitor) 30 min preop and for 10 consecutive days postop	**Reduced** mechanical hyperalgesia	(128)
	Spinal cord	TLR4	SMIR	Rats	**Increased** in ipsilateral DRG from 5 to 20 days	**i.t.** LPS-RS (TLR4 inhibitor) 30 min preop and for 10 consecutive days postop	**Reduced** mechanical hyperalgesia	(128)

CNS, central nervous system; DH, dorsal horn; DOR, δ opioid receptor; DRG, dorsal root ganglion; i.p., intraperitoneal; i.pl. intraplantar; i.t., intrathecal; KOR, κ opioid receptor; MOR, µ opioid receptor; PNS, peripheral nervous system; POD, postoperative day; Postop, postoperatively; Preop, preoperatively; s.c., subcutaneous; SMIR, skin and muscle incision retraction.

#### 4.1.1. Acute postsurgical pain

To understand the neurophysiological mechanisms involved in incision-induced postsurgical pain, the plantar incision model was developed by Brennan et al. (98). Under general anesthesia in rats, a 1 cm longitudinal incision is made through the skin, fascia, and plantar muscle, to extend towards the middle of the rat hindpaw. The skin is sutured, and the animals recover from anesthesia, after which pain behavior is measured from 1 h afterward. The Brennan model is an adequate recapitulation of tissue trauma experienced by patients undergoing surgery, due to the combination of trans-section of the skin, fascia and muscle retraction (1). Comparable to patients after surgery, temporary non-evoked guarding pain behaviors and longer-lasting pain behavior to punctate mechanical stimuli in rodent models are seen as proxy for non-evoked resting pain and evoked pain, respectively; and are hence pertinent to postsurgical pain in patients (130, 131). The plantar incision model was initially developed in the rat, but in 2003 this was extended to the mouse with a few modifications (i.e., 5 mm incision) and displayed similar results in behavioral pain studies (132). Mechanical hyperalgesia can be measured on the ipsilateral paw surrounding the incision (primary hyperalgesia) and in an area surrounding the injury (secondary hyperalgesia), for up to 6 days after incision (133). Additionally, heat hypersensitivity (but not cold) is robust at the site of incision and lasts for approximately 6–7 days (133). Since postsurgical pain is associated with anxiety and depression in patients, more recently studies have been underway to investigate anxiety- and depression-like behaviors after plantar incision. Rats have demonstrated an increase in both anxiety- and depression-like behaviors, and some reports show that anxiety-like behaviors lasted longer than hyperalgesia (134–136). An incision model in humans is also viable and may bridge the gap between animal studies and patients (137–139).

#### 4.1.2. Chronic postsurgical pain

The SMIR model was developed to mimic prolonged pain after a surgical incision that is comparable to patients (109). In this model, an incision (1.5–2 cm long) is made in the skin of the medial inner thigh and the superficial (gracilis) muscle layer is incised (7–10 mm) and is retracted with a microdissecting retractor for an hour, before suturing. Mechanical hyperalgesia lasts much longer than after plantar incision, for up to 3 weeks; however, heat hyperalgesia is not observed in this model. The SMIR model has been reproduced in a porcine model, which presents with numerous advantages (140). Notably, pigs are phylogenetically closer in proximity to humans in the sense so share similar metabolic pathways and comparable skin anatomy, including similar homology in wound healing compared to rodents (141). These features could be implemented for validating novel topical treatment options for postsurgical pain in patients. Many other pain models linked to surgical incision have been developed and are summarized in Figure 1. More recently, modified versions of the hyperalgesic priming paradigm (142) that use the Brennan hindpaw incision as a “priming” stimulus, followed by a second noxious insult to induce chronic pain [e.g., i.pl. PGE_2_ (143)], have been used to study the transition from acute to chronic postoperative pain. The use of perioperative opioids in rodents also precipitates persistent hypersensitivity after hindpaw incision (122), providing an ideal model for perioperative opioid-induced hypersensitivity observed in the clinic.

### 4.2. Challenges in translation

Specificity and clinically relevant behavioral outcome measures is a pertinent factor in defining the translational value of animal models of pain. Reflex-based behavior is one of the primary assessment methods to measure pain in rodents; nociceptive responses to pinprick and punctate stimuli are assessed in rodents frequently and similar stimuli are used to measure hyperalgesia near the surgical wound in patients (144). This provides some basis for parallels in mechanisms that are relevant across species (8, 145). Non-evoked guarding behaviors in rodents after incision may mirror non-evoked resting pain after surgery in humans (1). However, 44% of all studies published between 2014 and 2017 still assessed only one pain modality after incision, mainly including reflex responses (97). The use of multifaceted pain assays, such as gait analysis (146), locomotor function (147), and anxiety/depression-like behaviors (134) would be of great relevance to study the mechanisms for the transition from acute to chronic pain after surgery.

Another approach to aid translation of preclinical findings is to promote the use of large animal models, such as veterinary patients receiving surgery. Postsurgical pain models in the pig may be a useful alternative approach to induce experimental pain (140) as the skin of pigs is similar to human skin—a relatively thick epidermis, comparable wound healing processes, dermal blood vessel size, skin permeability, innervation of unmyelinated fibres and similar collagen make-up (141, 148, 149). The drawbacks to large animal models include difficulty in standardizing pain-related behavioral measures and cost. Beyond the nociceptive response, other physiological parameters such as inflammation, are monitored postoperatively in patients and could be back translated in preclinical models. Changes in inflammatory mediators in the wound vicinity have only recently been reported in the Brennan model, i.e., increased COX-2 activity and leukotriene B4 levels after incision (150). Furthermore, protein C, which is a natural anticoagulant, displayed antinociceptive activity (attenuation of guarding score) when injected into the wound and is a possible mediator of pain at rest after surgery in patients (150). Unbiased proteomic approaches to studying postsurgical pain are also helping to bridge the translational gap by identifying protein-protein interactions at the incision site that differ between human and mouse (151).

## 5. Cellular mechanisms of PPP

To date, findings from preclinical studies suggest that sensitisation of peripheral sensory neurons, neuroplasticity in the CNS, and neuro-immune interactions, are all potential mechanisms involved in the development and maintenance of PPP. Our understanding of these mechanisms predominantly stems from preclinical studies that focus on acute, subacute, and chronic incisional pain models in rodents (summarized in Table 3).

### 5.1. Peripheral sensitisation

Evidence for peripheral sensitisation in postsurgical pain derives from the use of the Brennan model of incisional pain in rodents, where typically, evoked pain behavior resolves by postoperative day (POD) 7 (98, 152). *In vivo* and *in vitro* electrophysiological data indicates that on POD 1, unmyelinated or poorly myelinated Aδ and C fibres display increased spontaneous activity and lower activation thresholds to heat and mechanical stimuli (153, 154). These findings correlate with both the non-evoked pain behaviors and mechanical and thermal hyperalgesia observed in behaving rodents. A number of pain mediators are thought to contribute to afferent sensitisation in the microenvironment of the incision site, which we describe in detail below.

#### 5.1.1. Ischemic-like pain mediators

Under ischemic conditions, tissue pH decreases, and lactate levels increase. The same is observed for several days after incision, with low tissue pH strongly correlated to pain behavior in rats that have received either an incision to the glabrous skin of the hindpaw, or the skin overlying the gastrocnemius muscle (155, 156). Surgically induced tissue ischemia was confirmed in further studies looking at tissue oxygen tension, which was significantly lower at the site of incision, and pimonidazole hydrochloride that was enhanced and is a marker for hypoxia (105). It is suggested that acid-sensing ion channel 3 (ASIC3) expressed by sensory neurons may be a sensor for hypoxia as it can detect lactic acid. Activation of ASIC channels on nociceptors generates ASIC currents, increasing the probability of action potential initiation, and neuron activation. Reducing ASIC3 activity with the antagonist APETx2 reduces thermal and spontaneous pain behaviors after incision in rats (112). Upregulation of ASIC3 in human chronic pain conditions has been described in biopsied samples from patients with Chron's disease, and is believed to contribute to visceral hypersensitivity in these patients (157). Whether or not it is upregulated at human incision sites remains to be determined.

#### 5.1.2. Neurotrophic factors

Neurotrophic factors such as nerve growth factor (NGF) and artemin are upregulated in skin and muscle tissue in as little as 1 h after plantar incision in rats, returning to baseline at POD 7, but are not upregulated in the DRG (117, 158). It is believed that fibroblasts and Schwann cells adjacent to the injury are responsible for NGF release (117, 159). Sequestering NGF with the use of systemically applied monoclonal antibody either before or after incision led to a reduction in heat hyperalgesia and non-evoked pain behavior, with no effect on mechanical sensitivity, suggesting different mechanisms of activity (121, 160). Indeed, when NGF interacts with its cognate receptor TrkA on nociceptors, the upregulation of the thermoreceptor TRPV1 is facilitated at the peripheral terminals of sensory neurons, causing heat hyperalgesia (161). NGF is therefore considered an important contributing factor to acute pain after incision, but evidence for its role in human postsurgical pain is limited.

#### 5.1.3. Cytokines

Several lines of evidence suggest that the cytokine IL-1 has an important role in the initiation and maintenance of incision-induced mechanical pain. Pro-inflammatory cytokine IL-1 is upregulated in the hindpaw tissue of rats after incision until pain behaviors resolve (117), IL-1 receptor knockout (KO) mice fail to develop mechanical hypersensitivity after injury, and the use of an IL-1 receptor antagonist either before or after incision ameliorated mechanical hypersensitivity (116). Indeed, the type 1 IL-1 receptor is highly expressed in a subpopulation of TRPV1+ nociceptors, conditional deletion of which also prevents the development of mechanical allodynia in a mouse model of RA (162). IL-6 is also upregulated in the skin and muscle of incised rats (117). It has previously been demonstrated that IL-6 causes protein synthesis in primary sensory neurons, which facilitates increased nociceptive activity (163) likely by upregulating expression of pain-related ion channels in nociceptors, such as TRPV1 (164). Furthermore, intraplantar (i.pl.) injection of IL-6, or plantar incision induces persistent nociceptive sensitisation to a subsequent i.pl. injection of PGE_2_, after the resolution of the initial hypersensitivity in a hyperalgesic priming paradigm (165). Mice were treated with local injections of the naturally occurring polyphenol, resveratrol, at the time of, and shortly after either injection of IL-6, or incision. Resveratrol increases AMP-activated protein kinase (AMPK), which inhibits ATP consuming processes such as protein translation largely due to inhibition of mammalian target of rapamycin (mTOR) signalling, and inhibition of mitogen activated protein kinase (MAPK) signalling (165). The study found that resveratrol dose-dependently attenuated acute pain caused by i.pl. IL-6 injection and incision and also prevented the transition to a chronic pain state when the animals were injected with PGE_2,_ potentially through prevention of IL-6-dependent protein synthesis in nociceptors (165). Targeting the IL-6-signalling pathway in rheumatoid arthritis (RA) patients with the use of the monoclonal antibody, tocilizumab, is analgesic (166). However, caution should be employed if targeting this cytokine for postsurgical pain, as IL-6 has an important role in wound healing, and RA patients undergoing tocilizumab treatment experience slower wound healing after surgery (167).

#### 5.1.4. Chemokines

Recent work has described the upregulation of the chemokines CCL17 and CCL22 by skin-resident dendritic cells and Langerhans cells in response to tissue injury caused by incision (114). This same study argued that their interaction with the cognate receptor, CCR4, on sensory neurons causes both mechanical and thermal hypersensitivity through CCL22, and only mechanical hypersensitivity with CCL17. CCR4 KO mice have significantly reduced acute postsurgical pain, as do control animals treated with either a CCR4 antagonist, or siRNA, or those depleted of dendritic cells. Furthermore, the group demonstrated that small diameter neurons in whole DRG cultures were able to produce action potentials in response to CCL22 application, with firing even greater in primary DRG cultures derived from animals that had been exposed to an incision. Whether this is due to a direct interaction between CCL22 and sensory neurons, or via a cell intermediate, remains to be determined.

The chemokine CXCL1/KC and its receptors CXCR1/CXCR2 also play a role in postsurgical pain, by recruiting inflammatory neutrophils to the incised paw. Neutrophil depletion reduced mechanical hyperalgesia after incision (120). Indeed, the *cxcl1* and *cxcl2* gene were reported as the most rapidly expressed genes (1 h) at the site of incision in a recent unbiased cell sequencing study (168). Other studies indicate a role for neutrophils in chronic pain conditions, including fibromyalgia (169), although how they activate or sensitise nociceptors has not been resolved.

#### 5.1.5. Complement

Complement C5a also contributes to incisional pain (113). Hindpaw incision causes increased expression of C5 and C5aR mRNA, and C5a protein in the skin (but not the DRG or spinal cord) for at least 72 h after incision. Local administration of the C5aR antagonist PMX53 via i.pl. injection before and after incision significantly attenuated thermal and mechanical hypersensitivity. Thus, high local concentrations of C5a produced in wounds likely contributes to postsurgical pain. Further work by the same group demonstrated that global deletion of C5aR in mice reduces thermal and mechanical hypersensitivity, and inflammatory responses (including IL-1β and NGF expression, and neutrophil infiltration at the wound site) after hindpaw incision (170).

The presence of the above mediators in the wound environment causes an afferent barrage from sensitized nociceptors to the spinal cord, initiating the process of central sensitisation.

### 5.2. Central sensitisation

Central sensitisation refers to the amplification of central pain pathways and is a form of long-term adaptive neuroplasticity. It is responsible not only for enhanced behavioral sensitivity (hyperalgesia), but also spreading of the sensitized area (171, 172). Evidence for postsurgical central sensitisation is observed in rodent neurophysiological studies *in vivo*, which have demonstrated increased activity in the dorsal horn (DH) neurons of animals on POD 1. This is observed as spontaneous activity, expansion of the receptive field, and enhanced activity in response to mechanical stimulation (173, 174). Sensitisation in the DH can be reversed by blockade of afferent input indicating that peripheral drive is essential at this stage for central sensitisation. We refer primarily to spinal sensitisation within this review, but several studies have also focussed on brain neuroplasticity in postsurgical pain (175).

#### 5.2.1. AMPA receptors

In contrast to inflammatory and neuropathic pain, the initial stages of incision-induced spinal sensitisation are believed to be NMDA-independent. Early after incision, the NMDA antagonist dizocilpine (MK-801) fails to block DH sensitisation, unlike the non-NMDA ionotropic EAA receptor antagonist, NBQX [2,3-dioxo-6-nitro-7-sulfamoyl-benzo(f)quinoxaline] (176). The latter finding indicates a role for AMPA receptors in spinal sensitisation after incision, which has been confirmed by further pharmacological studies (110). Further mechanistic insight shows hindpaw incision leads to phosphorylation of the GluR1 subunit of the AMPA receptor at serine-831 by protein kinase C gamma (PKCγ) (123) This leads to increased insertion of Ca^2+^ permeable AMPA receptors to the neuronal plasma membrane, enhancing neuronal activity and DH excitability. Intrathecal (i.t.) pre-treatment of rats with small interfering siRNA targeting PKC reduced pain hypersensitivity 3 h after incision. Further evidence for the role of the GluR1 subunit in acute incisional pain is observed when the transmembrane protein stargazin, responsible for regulating synaptic targeting of AMPA receptors, is pharmacologically down-regulated in a rat incision model, preventing an incision-induced increase in membrane GluR1 in the ipsilateral DH, as well as decreasing postsurgical pain at 3 h (115). Therefore, spinal sensitisation after plantar incision is initially maintained by the barrage of peripheral input from nociceptors, as well as non-NMDA/AMPA receptors.

### 5.3. Epigenetic changes

Epigenetic events include modification of histone proteins, microRNA expression, and changes in global DNA methylation, which have all been demonstrated to occur after hindpaw incision in rodents. Blockade of DNA methyltransferase (DNMT) in mice leads to a reduction in incision-induced thermal and mechanical hyperalgesia and in oedema (177). The identified methylation target was the *μ*-opioid receptor gene *Oprm1,* which has increased expression in the skin following hindpaw incision (but not the spinal cord or DRG) at POD 1 and 3, and was increased further at POD 1 when DNMT was inhibited with 5-AZA-CdR (5-Aza-2′-deoxycytidine).

Additionally, incisional pain in rodents is associated with enhanced *Bdnf* [brain-derived neurotrophic factor, the agonist for tyrosine kinase receptor B(trkB)] and *Pdyn* (prodynorphin, the precursor to the k-opioid agonist, dynorphin) expression in the spinal cord, at POD 1 and 3, respectively (178). Chromatin immunoprecipitation assays demonstrated that *Bdnf* and *Pdyn* promotors were more strongly associated with acetylated H3K9 after morphine administration and incision (rather than either alone). I.t. injection of the TrkB antagonist ANA-12, and the *κ*-opioid receptor (KOR) selective antagonist norbinaltorphimine (nor-BNI) both reduced hyperalgesia on POD 1 or 3; however, when used individually only nor-BNI reduced hyperalgesia on POD 3. Co-administration of histone acetyltransferase inhibitor anacardic acid daily attenuated incision enhanced hyperalgesia in morphine treated mice. Therefore, both the BDNF-TrkB and dynorphin-KOR systems may be accessible therapeutic targets for postsurgical pain (178).

### 5.4. Latent sensitisation

“Latent sensitisation” refers to a phenomenon of long-lasting pain vulnerability after opioid exposure, stress, or tissue damage (179). Opioid-dependent analgesic systems are thought to be an adaptive response to surgery which allows for natural recovery to acute surgical pain, masking a heightened state of sensitisation (180). Pain can be “unmasked” by opioid inverse agonists/antagonists to precipitate hyperalgesia after the resolution of tissue injury-induced hypersensitivity. In one study where mice received a plantar incision and pain behavior was monitored up until POD 20 when mechanical sensitivity had returned to baseline, animals received naloxone or the KOR selective antagonist nor-BNI on POD 21 and developed a hypersensitivity comparable to POD 2—also shown to be a NMDA receptor-dependent process (126). Interestingly, peripherally restricted naloxone methiodide did not produce hyperalgesia to the same extent as naloxone, indicative of a central role for KOR. Other groups have confirmed this finding, and that tonic endogenous KOR-mediated inhibition of latent sensitisation is more pronounced in females. μ-opioid receptors (MOR) also contribute equally in both sexes to this phenomenon (125). Latent sensitisation (induced by mild heat injury) has also been described in humans, although there was heterogeneity between volunteers suggesting individual differences in the adaptive opioid response (181). It is hypothesized that the inability of a patient to engage this endogenous analgesia may be a predictor of the likelihood of a patient to go onto developing chronic postsurgical pain, indeed, those that have less efficient diffuse noxious inhibitory control (DNIC), are more likely to develop chronic post-thoracotomy pain (182). While the contribution of supraspinal structures to latent sensitization is out of the scope of this review, we direct the reader to recent studies addressing these (124, 183).

### 5.5. Hyperalgesic priming

The hyperalgesic priming paradigm can be used in rodents to reproduce PPP (143, 184). For example, following the Brennan hindpaw incision and resolution of mechanical allodynia by POD 13, mice receive an i.pl. injection of PGE_2_ into the same paw to precipitate persistent hypersensitivity. By using hyperalgesic priming, Asiedu and colleagues describe a mechanism of central sensitisation responsible for the maintenance of incisional pain (127). Protein kinase M ζ (PKMζ), is essential for supraspinal late long-term potentiation (LTP) and PKMζ inhibition leads to the loss of previously established memories (185). Thus, the group hypothesized that PKMζ is responsible for promoting a sensitized state in the DH after the induction of PPP in rodents, by the formation of a so called “pain memory” at the level of the spinal cord. Indeed, they found that inhibiting this pathway with an i.t. injection of the PKMζ inhibitor ZIP prior to PGE_2_ injection ameliorated the persistent allodynia induced by PGE_2_ (127). Another group went on to explore this further by using a modified form of the above model, injecting carrageenan instead of PGE_2_, prior to incision (186). Treatment with ZIP prior to incision attenuated hypersensitivity, again indicating a spinal PKMζ-dependent mechanism.

### 5.6. Opioid-induced hyperalgesia (OIH)

Sustained opioid use can lead to postsurgical OIH in humans (187) and in rodent models of postsurgical pain (188). Although opioids remain the analgesic of choice for acute pain management, caution must be taken with over prescription as they can facilitate postsurgical pain in patients. One of the mechanisms is thought to involve NMDA receptors; as studies using perioperative ketamine, an NMDA receptor antagonist, have demonstrated that ketamine prevents enhanced postsurgical pain in fentanyl treated rats and can improve pain management with exogenous opioids (122). Thus, ketamine can prevent central sensitisation that is triggered by nociceptive input (surgery) and exaggerated by fentanyl.

The use of pre-clinical models of incisional pain enables a better understanding of the mechanisms that underpin the development and maintenance of PPP. This, alongside unbiased transcriptomic (168) and proteomic studies (151) is essential to identifying novel targets for its treatment, as the efficacy of current clinical interventions for PPP is limited and will be discussed in the following sections.

## 6. Prevention and treatment of PPP

Effective translation of basic and clinical scientific findings into clinical practice remains the major hurdle to prevention and/or adequate treatment of PPP. Advancements in surgical technique such as laparoscopic or thoracoscopic access do not necessarily prevent the development of PPP in all patients (189, 190), and analgesic treatments are not targeted to specific aspects of postsurgical pain, such as movement-evoked pain or adequate wound healing. With risk factors such as the invasiveness of surgery and consequent tissue damage, as well as the severity of acute postsurgical pain, PPP offers a unique interventional window during the pre- intra- and immediately post-operative periods.

### 6.1. Optimizing surgical approach

One putative risk factor for PPP is the extent of tissue damage during surgery, including damage to nerves. The latter can be minimized by identifying and preserving these during surgery and has been shown to reduce the development of chronic pain after inguinal hernia repair (191). There is also some evidence to suggest that minimally invasive surgery, such as laparoscopic surgery can lead to less persistent pain and numbness with this particular type of operation (192). However, a more recent meta-analysis argued against this instead suggesting that there was no difference between chronic pain in patients that underwent either laparoscopic or open surgery for an inguinal hernia repair, despite positively impacting acute postsurgical pain (193). As both reviews highlighted, one of the downsides of laparoscopic surgery is the increased operating time, as well as an increased incidence of serious complications such as visceral and vascular injury. Importantly, the duration of surgery is a risk factor for PPP (although this may be due to increased complexity of pathology, and/or intraoperative tissue damage) (92).

In the case of other procedures such as hysterectomy, studies have reported that a laparoscopic approach, rather than vaginal, reduces acute pain without impacting on the prevalence of chronic pain (194). Despite this, the manner of wound closure after hysterectomy can impact upon the development of PPP, with closure of the parietal peritoneum increasing its incidence (195).

Performing less extensive surgery may reduce the incidence of PPP for some interventions. Post-mastectomy PPP manifests as pain localized to the axilla, the medial upper arm, and/or the anterior chest wall on the affected side. In the case of axillary node dissection, this may be caused by damage to the intercostobrachial nerve. Other nerves including the medial and lateral pectoral, long thoracic, or thoracodorsal nerves may also be damaged during breast cancer surgery and can contribute to neuropathic pain. It is possible to prevent damage to the intercostobrachial nerve by performing a sentinel lymph node biopsy during a lumpectomy or mastectomy, which, depending on the outcome, may prevent unnecessary axillary dissection (196).

In the case of thoracotomy (a form of surgery with the highest incidence of PPP), there are many surgical variables linked to the development of chronic pain (76). These include the types of incision for an open procedure (rib resection vs. retraction), as well as the extent of intercostal nerve preservation. It has been demonstrated that resection rather than retraction of the ribs causes less damage to the intercostal nerve, thus reducing the incidence of PPP (197). In a recent meta-analysis, the use of video-assisted thoracoscopic surgery vs. open thoracotomy was shown to also reduce the incidence of PPP at 6 months post-surgery (198). Efforts to reduce the invasiveness of surgery also extend to orthopaedic surgery, in an attempt to reduce tissue and nerve damage, as this may lead to PPP (199).

Nevertheless, there is still insufficient evidence to recommend a definitive surgical technique to prevent the development of PPP. On the other hand, risk may be minimized by avoiding damage to local nerves, minimizing the duration of surgery, choosing a minimally invasive procedure, and avoiding extensive surgery where possible.

### 6.2. Regional analgesia

A major predictor of PPP is the presence of pain before surgery, and the extent and duration of acute postsurgical pain. Pain management strategies include the use of regional anesthesia before, during and/or soon after surgery. Cochrane Systematic review and meta-analysis pooling results from surgeries with the highest rate of PPP (breast surgery, thoracotomy and limb amputation) found that regional anesthesia with local anesthesia led to a reduction in the development of PPP, likely through preventing and/or minimizing peripheral sensitisation and the subsequent development of central sensitisation (200). This was demonstrated in patients receiving an epidural prior to undergoing thoracotomy, or those that received an infusion of intravenous (i.v.) local anesthetics for breast cancer surgery. Although results could not be synthesized from limb amputation due to differences in the mode of treatment or in the reporting of results, an example of successful regional anesthesia during limb amputation found that the use of optimized epidural analgesia or intravenous PCA (patient-controlled analgesia), starting 48 h preoperatively and continuing for 48 h postoperatively, significantly decreased phantom limb pain after 6 months in limb amputees, compared to those receiving conventional analgesia (201).

### 6.3. Preventative pharmacotherapies

#### 6.3.1. Ketamine

The development of PPP may also be prevented with the use of preventative pharmacotherapies. Preclinical studies suggest that perioperative ketamine may be effective in reducing opioid consumption and acute postsurgical pain (122), but a meta-analysis has reported that the use of perioperative ketamine for analgesia in humans has so far proven inconclusive (202), although may be useful in reducing opioid consumption and pain intensity in the postoperative period in patients taking opioids prior to surgery (203). Despite this, an earlier systematic review found that by separating studies depending on the route of ketamine delivery, those that received i.v. ketamine reported a statistically significant reduction in the prevalence PPP at 3- and 6-months post-surgery (204). These contradictory findings and clinical equipoise led to a large multicentre Phase 3 randomized clinical trial with the primary aim of “reduction of chronic postsurgical pain with ketamine” (ROCKet trial, registration: ACTRN12617001619336). Ketamine will be administered as an i.v. infusion intraoperatively and continued postoperatively for a maximum of 72 h. The primary endpoint is the incidence of PPP reported by the patient at telephone follow-up, 12 months after surgery (205). Ketamine is generally well-tolerated in patients, however, there are a number of common adverse effects associated with its use including headaches, dizziness, and drowsiness limited to the duration of administration and shortly after (206).

GabapentinoidsMany incidences of PPP include an element of neuropathic pain that can be measured using standardized questionnaires (e.g., DN4) and bedside sensory testing for allodynia (87). Anticonvulsants such as pregabalin and gabapentin, which are indicated as first or second line analgesics for pain relief from neuropathy, have also been trialled for the prevention of PPP. These drugs bind to the alpha-2-delta subunit of pre-synaptic voltage-gated calcium channels and inhibit calcium influx. This in turn attenuates glutamate release therefore reducing pain transmission and central sensitisation (207). The introduction of gabapentinoid use perioperatively was initially met with enthusiasm, with an early study describing a single preoperative dose of gabapentin having an analgesic effect 4 h post-surgery. Studies such as these, on the backdrop of the opioid crisis, have led to the wide use of gabapentinoids in an attempt to decrease opioid consumption for pain relief in the postoperative period (208). However, recommendations for the use of gabapentinoids in the management of postsurgical pain are inconsistent, and increasingly emphasise the potential for adverse events, such as risk of abuse, dizziness, and respiratory depression (209). Consequently, the former American Pain Society and European Society of Regional Anesthesia pain therapy guidelines offer conflicting recommendations for the use of gabapentinoids in the perioperative period. A meta-analysis including 281 trials has reported no meaningful analgesic effect of gabapentinoids in comparison to placebo on acute or chronic postsurgical pain. Furthermore, while preventing vomiting and nausea, adverse events such a dizziness and visual disturbance were increased postoperatively (209). Further well-controlled trials are recommended for prolonged perioperative use following specific surgical incisions [sternotomy for cardiac surgery (202)].

#### 6.3.2. Lidocaine

Systemic administration of the sodium channel blocker lidocaine has been tested for its capacity to prevent of PPP (202). Clinical trials so far have been cautiously optimistic for the use of i.v. lidocaine during surgical interventions as a preventative intervention and further evidence is needed to validate the efficacy of this approach, including adverse effects (202).

#### 6.3.3. NSAIDs

Non-steroidal anti-inflammatory drugs (NSAIDs) block cyclo-oxygenase (COX) enzymes and inhibit the production of prostaglandins, resulting in an anti-inflammatory effect and analgesia (210). Positive effects in the prevention of PPP have been reported in a recent meta-analysis (202). A statistically significant treatment effect was observed in patients 12 months after surgery, when drugs were administered for 24 h or less, but not in those that had received treatment for more than 24 h, at either 3, 6, or 12 months post-surgery, indicating the duration of treatment as an important factor. NSAIDs are considered safe with minimal adverse effects, although caution should be used when prescribing to patients with underlying chronic kidney disease, a cardiac history, or gastrointestinal complications (211).

#### 6.3.4. Opioids

Opioids remain the gold standard for pain relief for moderate to severe perioperative pain. Achieving adequate pain control with opioids is an established approach for preventing persistent post-thoracotomy pain (212) and phantom limb pain (201). However, their use should be approached with caution not only due to serious side effects including sedation, respiratory depression, dependence and tolerance, but also due to the risk of developing OIH, as may be the case with remifentanil use at high doses intraoperatively (212, 213).

#### 6.3.5. Multimodal anesthesia

The use of multimodal anesthesia during the perioperative period has also been demonstrated to be effective in the prevention of PPP, i.e., a combination of agents that have different mechanisms of analgesia that may have a synergistic effect by modulating pain signals at various points of the pain pathway, rather than using a single drug. This can include combinations of gabapentinoids, NSAIDs, acetaminophen as well as regional anesthesia (214). There are few studies evaluating the efficacy of multimodal analgesia on PPP, however an example of its effectiveness can be seen in a breast cancer surgery trial, whereby pain and analgesic consumption was significantly reduced at 3 months in patients that received gabapentin, a mixture of local anesthetic cream, and ropivacaine wound infiltration (215). Pre-clinical models demonstrate analgesic synergy between clinically approved analgesics, as well as novel pharmacotherapies including a combination of selective Na_v_1.7 inhibitors, with baclofen or opioids (124, 216). A detailed summary of randomized controlled trials using pharmacological interventions to prevent the development of PPP can be found in the following review (202).

## 7. Management of PPP

Many of the interventions used to treat pain in the acute postoperative period continue to be used for the management of PPP. In the first instance, it is important to identify the aetiology and type of pain if PPP is suspected, and to exclude recurrent malignancy in the case of cancer surgery, or to exclude other postoperative complications such as infection. Strong opioid use is to be avoided at this stage to avoid risk of dependency, which highlights the need for novel analgesics.

### 7.1. Pharmacotherapies

Current pharmacotherapies for the management of PPP include those used in neuropathic pain such as the anticonvulsants (gabapentinoids), tricyclic antidepressants (amitriptyline and nortriptyline), serotonin-norepinephrine reuptake inhibitors (duloxetine and venlafaxine), and topical lidocaine or capsaicin as a first line of defence, the studies of which have recently been summarized (217). However, a 2017 meta-analysis of the available randomized trials for PPP pharmacological treatment revealed that most pharmacological interventions tested were in isolation and did not include multimodal or multidisciplinary treatment programmes (218). Furthermore, there was no sufficient data to conclude on effectiveness or safety.

### 7.2. Nerve block

For patients whose pain levels are not responsive to pharmacotherapy, interventions such as nerve blocks, nerve ablation and neuromodulation can also be used. In the case of sternotomy-induced neuralgia, successful treatment was achieved using repeated bupivacaine blocks, phenol blocks or alcohol blocks (219). Furthermore, epidural injections have proven to provide pain relief for lumbar or cervical post-surgery syndrome (220).

Nerve entrapment or damage of nerves in the abdominal wall is often the cause of chronic abdominal wall pain post-surgery. One case report describes neural blockade of the thoracolumbar nerves supplying the anterior abdominal wall by temporary local infusion of anesthetic agents producing long-term pain relief, thereby suggesting a peripheral source for the abdominal pain (221). Furthermore, axillary brachial plexus block in a patient suffering from complex regional pain syndrome type I (CRPS I) of the upper limb after surgical release of carpal tunnel syndrome on the right hand, successfully alleviated symptoms, when combined with patient-controlled analgesia (PCA) (222). Other neural blocks include the use of botulinum toxin injections, which when injected into painful areas of chronic post-thoracotomy pain can provide significant pain relief (223), as well as the abdominal wall after laparoscopic ventral hernia repair (224). Additionally, injection or infusion of lidocaine into the DRG transiently relieved stump or phantom limb pain (PLP) (225).

More recently, novel ultrasound guided blocks have proven effective in the management of PPP after breast and abdominal surgery. In pilot studies following breast surgery, either intercostobrachial or pectoral nerve block with bupivacaine, may provide pain relief (226, 227). For patients suffering with surgery-induced chronic abdominal wall pain, ultrasound-guided transverse abdominis plan (TAP) block with local anesthetics provided significant pain relief (228, 229)

### 7.3. Nerve ablation

Where phantom limb sensation is useful for prosthesis, stump pain or PLP can be a debilitating disorder and afflicts almost 60% of amputee patients 1 year post-surgery (82). Amputation directly affects the peripheral nervous system (PNS), which in turn causes alterations to the CNS due to changes in sensory and movement signalling. Whereas pharmacotherapy may potentially be useful in treating PLP, such as gabapentinoids to target the neuropathic component of the disease (230), nerve ablation techniques such as phenol injections or radiofrequency ablation of stump neuromas or DRG, have been found effective. Radiofrequency ablation of the DRG or dorsal root entry zone (DREZ) shows potential for the treatment of chronic ilioinguinal pain following inguinal hernia repair as well for chronic post-thoracotomy pain (231).

Other methods of PPP management include surgical resection in the case of neuroma formation, or physical therapies such as massage, physiotherapy and acupuncture. Psychological interventions, for example cognitive behavioral therapy, have also been found useful in managing chronic pain. Due to the complexity of PPP, an individualized patient-centred approach is essential in its prevention and treatment. High quality randomized trials including multimodal interventions are required to validate and build upon positive outcomes described in the trials above.

## 8. Conclusion

Chronic pain after surgery is a significant clinical problem that affects approximately 10%–50% of patients worldwide. Over the last decade improvement in study design has enabled better stratification of patients according to their risk of developing PPP, but the efficacy of current treatments for acute and chronic postsurgical pain remains contentious and clinical trials often focus on individual, as opposed to multimodal, treatment strategies. Contradictory reports on therapeutic efficacy of these approaches emphasise the need for large-scale homogeneous replication studies over different study sites in order to minimise variability and validate findings. Where current treatments may prove unsuccessful, studies in pre-clinical models of acute and persistent pain due to a surgical intervention continue to provide novel therapeutic targets for clinical benefit.
